# The Pentameric Channel of COMPcc in Complex with Different Fatty Acids

**DOI:** 10.1371/journal.pone.0048130

**Published:** 2012-11-02

**Authors:** Ainsley A. MacFarlane, George Orriss, Natalie Okun, Markus Meier, Thomas Klonisch, Mazdak Khajehpour, Jörg Stetefeld

**Affiliations:** 1 Department of Chemistry, University of Manitoba, Canada; 2 Human Anatomy & Cell Science, University of Manitoba, Canada; 3 Department of Chemistry, Microbiology, Biochemistry and Medical Genetics, University of Manitoba, Canada; Russian Academy of Sciences, Institute for Biological Instrumentation, Russian Federation

## Abstract

**Background:**

COMPcc forms a pentameric left-handed coiled coil that is known to bind hydrophilic signaling molecules such as vitamin D_3_, and vitamin A.

**Principal Findings:**

In an integrated approach we reveal the unique binding properties of COMPcc for saturated and unsaturated fatty acids. Our observations suggest that residues Met33 (gating pore), Thr40/Asn41 (water chamber) and Gln54 (electrostatic trap) are key elements for the binding of fatty acids by COMPcc. In addition, this work characterizes the binding of various fatty acids to COMPcc using fluorescence spectroscopy. Our findings reveal a binding trend within the hydrophobic channel of COMPcc, namely, that is driven by length of the methylene tail and incorporation of unsaturation.

**Conclusion/Significance:**

The unique binding properties imply that COMPcc may be involved in signalling functions in which hydrophilic ligands are involved. The pentameric channel is a unique carrier for lipophilic compounds. This opens the exciting possibility that COMPcc could be developed as a targeted drug delivery system.

## Introduction

**Figure 1:**
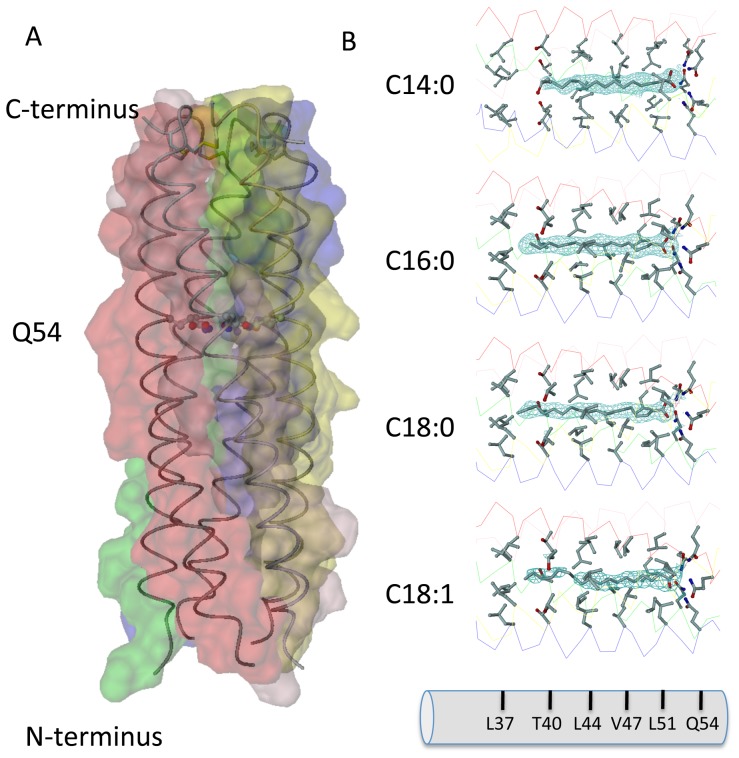
The COMPcc channel in complex with fatty acids. (**A**) The pentameric COMPcc channel is drawn as Cα-backbone superimposed by van der Waals spheres of individual chains that are highlighted by different color schemes. Both N –and C-termini as well as the Q54 ring system (drawn in stick-and ball mode) are labelled. (**B**) The individual COMPcc - fatty acid complex structures show that one molecule of each fatty acid is bound in the N-terminal cavity of the hydrophobic channel (the C-terminal part of the structure is not hown). The aliphatic side chains of individual knobs-into holes residues (Leu37, Thr40, Leu44, Val47, Leu51 and Gln54) inside of the coiled coil cause 6 regular constrictions to the diameter of the pore, varying in size between 2 and 6 Å as defined by the van der Waals radii. Individual simulated annealing 2Fo-Fc-omit maps (1.2σ contour level) superimposed on: myristic acid (C14:0), palmitic acid (C16:0), stearic acid (C18:0) and oleic acid (C18:1) are drawn in lightblue. Water molecules are not shown for clarity reasons (see in detail Figures 2 and 4, instead).

Cartilage oligomerization matrix protein (COMP) is a non-collagenous glycoprotein of the thrombospondin family that is found in cartilage [1], tendons [2], [3], and ligaments [4]. It is a homopentamer consisting of five subunits held together by interchain disulfide bridges in the N-terminal coiled-coil domain (COMPcc) composed of residues 27–72 (Fig. 1A). The COMPcc chain fragment forms a parallel left-handed coiled-coil with an average length of 70 Å and an average outer diameter of about 30 Å. The axial pore of the pentamer is divided by the hydrophilic Gln54 ring system into two hydrophobic cavities that are exclusively lined with aliphatic side chains [5], [6]. According to the heptad repeat pattern of left-handed coiled coils, residues in *a* positions of COMPcc form perpendicular knobs-into holes, whereas residues in *d* position are oriented in a parallel manner [7]. The binding of a number of biologically relevant hydrophobic compounds to recombinantly expressed COMPcc has been shown, with crystal structures available for the COMPcc-vitamin D_3_ [8], COMPcc-all-*trans* retinol, and COMPcc-benzene complexes [9]. The binding properties of the hydrophobic channel suggest the potential of COMPcc to be used as a storage and delivery system for hydrophobic compounds [10].

Fatty acids (FA's) have diverse and important biological functions in cells. They are involved in protein acylation, transcription regulation, apoptosis, energy production and storage, and membrane synthesis [11], [12]. They are essential key components in numerous signaling cascades involving TLR and insulin signaling as well as inflammatory responses [12], [13]. FA's comprise approximately 30–40% of total fatty acids in animal tissues, with the majority being palmitic acid (15–25%), followed by stearic acid (10–20%), myristic acid (0.5–1%), and lauric acid (<0.5%) [14]. Natural receptors for FA's include family members of the albumin and fatty acid-binding protein (FABP) family [15]. These proteins serve to increase the solubility of fatty acids and mediate their transport within cells. While there are many members of the FABP family with a great deal of variance in protein sequence, all members share a common ß-barrel structural motif [15]. The 10-stranded antiparallel ß-barrel contains a hydrophobic core to which fatty acids bind. The core is capped on one end by an N-terminal helix-turn-helix motif. Inside the binding pocket, the carboxyl group is coordinated through electrostatic interactions with tyrosine and two arginine residues. The hydrocarbon tail is oriented with hydrophobic residues on one side and ordered water molecules on the other side [16]. Multiple fatty acid binding sites have been shown for Human Serum Albumin revealing a combined contribution of electrostatic and hydrophobic forces to the binding interactions [17]. Interestingly, the carboxylate head group of the bound fatty acids are more tightly bound than their methylene tail [18].

In the current work, we have solved the crystal structures of COMPcc in complex with myristic acid (C14:0), palmitic acid (C16:0), stearic acid (C18:0) and oleic acid (C18:1). In addition, the binding of these ligands to COMPcc in solution has also been studied with fluorescence spectroscopy. From the binding constants we have deciphered a trend in binding favorability that is determined by length of the aliphatic tail and geometry altered by introduction of a *cis*-configured double bond. A significant finding of this study is the observation that only fatty acids in an elongated configuration can pass the selectivity filter formed by the ring of five Met33 residues located at the entrance to the hydrophobic channel.

## Materials and Methods

### Expression and purification of recombinant COMPcc

The coiled-coil domain of rat COMPcc comprising residues 27–72 was prepared as described previously [5]. Purified COMPcc was dialyzed against PBS pH 7.4 and concentrated to 10 mg/mL using an 10 kDa Amicon membrane (Millipore).

### Crystallization and Data Collection

Crystallization experiments were performed at room temperature employing the vapour diffusion technique. Hanging droplets were made by mixing 2 µl protein solution (10 mg/ml) with 0.2 M sodium acetate, 0.1 M HEPES, pH 7.4 and 2 M ammonium sulfate. Individual fatty acids obtained from Sigma were soaked in an equimolar ratio into the crystals for 6 hours. Palmititc acid titration experiments were performed by adding molar excess and incubation overnight. The crystals belong to spacegroup P2_1_ and contain one molecule of the pentameric COMPcc within the asymmetric unit. To analyze the influence of different effectors (pH, ions and organic solvents) four crystal structures performing different crystallization conditions were determined (data not shown). The high resolution data sets were collected at synchrotron CLS (PX-Beamline) on a MAR research imaging plate detector. Diffraction images were processed using program suite MOSFLM [19] and the structure factors were scaled and reduced using SCALA from the CCP4 package [20]. Statistics of the merged data is given.

### Structure determination and refinement

Molecular replacement was performed using the AMORE program of the CCP4 package [20]. A Poly-serine model of native COMPcc structure (PDB-code:1MZ9) was used as search template. Positional refinement was performed with CNS using the maximum likelihood method [21]. Five to ten percent of the reflections were excluded for use in a cross validation set. Refinement with CNS was alternated with manual electron density refitting of side-chains and terminal regions using MAIN. At this stage the individual fatty acid molecules have been fitted into a 3.0σ contoured Fo-Fc difference map. To determine the favoured axial orientation of the ligands within the pentameric channel a 2° stepwise refinement (conjugated gradient minimization together with individual B-factor refinement) along the five-fold local symmetry axis was performed [22]. Interpretation of the electron density maps for each solution together with monitoring of the R_free_/R_value_ ratio revealed that no preferred orientation can be detected. In further refinement overall anisotropic B-factor and bulk solvent corrections were utilized. Simulated annealing omit maps confirmed the correctness of the protein and ligand structures. Water molecules were added chosen by distance criteria and hydrogen bonding geometry and were tested for position in spherical density, reasonable temperature factors, real space R-values, and improvement of the R-factors. The program CHARMM [23] and the effective energy function EEF [24] were employed for ⊗G determinations as described previously [25]

### Fluorescence spectroscopy of COMPcc-fatty acid complexes

Steady-state fluorescence spectra were measured on a Fluorolog-3 Horiba Jobin Yvon spectrofluorometer (Edison, NJ). The sample was held in a 10×10 mm quartz cuvette equipped with a continuous stirrer. The data were analyzed with Sigmaplot (Point Richmond, CA) software. The reaction was thermostatically controlled at 25°C by a Jeio-Tech refrigerating bath circulator (Des Plaines, IL). All fluorescence data were collected in 1× PBS buffer, pH 7.4. From the fluorescence enhancement profile of CPA, the fraction of ligand bound protein (*f*) can be calculated using:
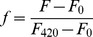
(1)where F_0_ is the fluorescence of protein sample when no CPA has been added, F is the protein fluorescence at any given CPA concentration and F_420_ is the protein fluorescence in the presence of 3 µM of CPA. In the case of one ligand binding site, *f* follows a hyperbolic dependence upon ligand concentration given by:
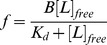
(2)where *B* is a constant, K_d_ is the dissociation constant and [*L*]*_free_* is the concentration of free ligand (in this case CPA). The data in Fig. 3B show a good hyperbolic correlation. Therefore, the binding of CPA to COMPcc is consistent with hyperbolic one site binding and the experimentally determined binding constant was 0.7±0.1 µM.

The probe CPA can also be used to characterize the binding of other fatty acids to COMPcc. The addition of fatty acids (FA) to the CPA-COMPcc complex will displace CPA leading to a decrease in fluorescence. If the concentrations of COMPcc and CPA are kept significantly lower than the K_d_ value, the following dissociation constants can be defined for the CPA-COMPcc and FA-COMPcc complexes:
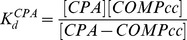
(3)and
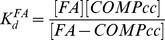
(4)In these experiments, the concentration of COMPcc is kept low, both relative to K_d_^CPA^ and relative to CPA and the concentration of CPA is also kept significantly lower than K_d_^CPA^ (the initial concentration of COMPcc is ∼0.1 µM and CPA is equal to 0.4 µM). In the case where there is no FA added the concentrations are [*COMPcc*] = [*COMPcc*]_0_ and [*CPA*]≈[*CPA*]_0_ = 0.4 µM. In this case the concentration of the CPA-COMPcc complex will be equal to:
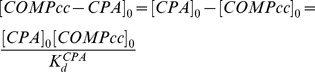
(5)At 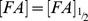,the concentration of the COMPcc-CPA complex is equal to one half the initial:
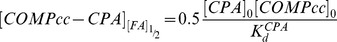
(6)Writing a mass balance for COMPcc we obtain:
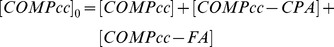
(7)When 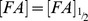:
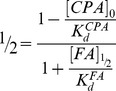
(8)The dissociation constant 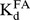 can be calculated using the value of [*FA*]_1/2_ (the amount of fatty acid that reduces the CPA fluorescence to half its original value.

## Results

### X-Ray structures of the individual COMPcc-fatty acid complexes

**Figure 2:**
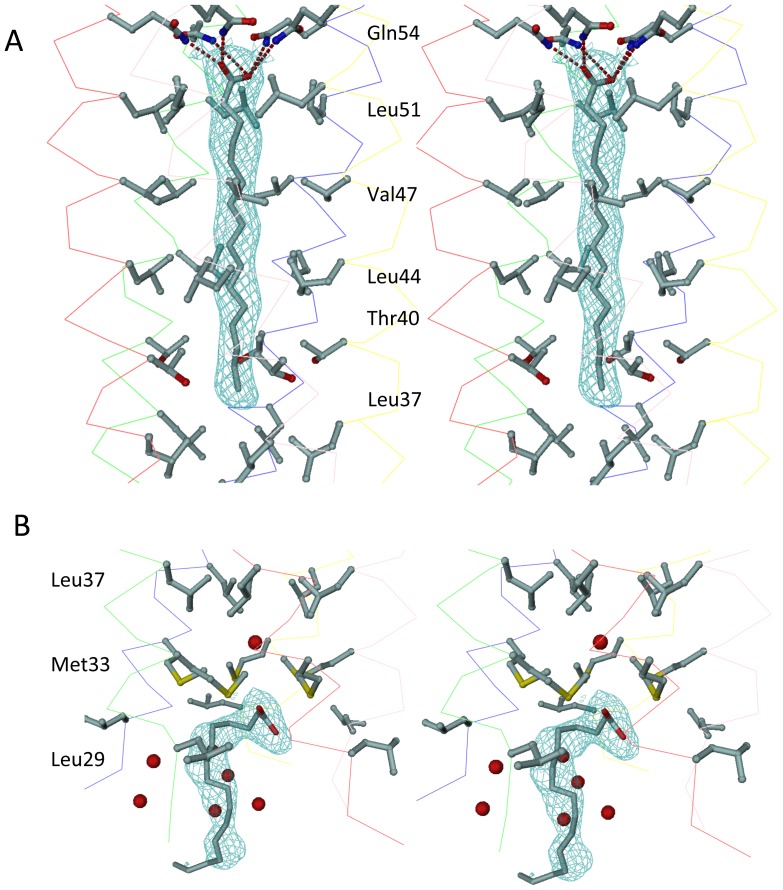
Stereoview of the 2.2 Å resolution 2Fo-Fc omit map (1.2σ contour level) of palmitic acid inside (A) and outside (B) the pentameric channel. The electrostatic fixation of the carboxylate head group of palmitic acid inside the channel (A) is symbolized by red dotted lines. Individual side chains are labelled. Water molecules are drawn as red spheres.

The coiled-coil domain of COMP comprising residues 20–72 was obtained by recombinant expression in *E. coli* as described previously (see also Materials and Methods and [8]). The individual crystal structures of the COMPcc-fatty acid complexes were solved by molecular replacement using the apo-COMPcc version (PDB code:1MZ9) as a search template (Fig. 1; see also Table 1). In the individual COMPcc-fatty acid complex structures, one molecule of the respective fatty acid is bound inside the N-terminal hydrophobic compartment in a linear, elongated conformation. The longitudinal axis of the fatty acids are parallel to the five-fold channel symmetry (Fig. 1B). Diffusion of the lipophilic ligands into the channel likely occurs through the N-terminus. Additional electron density in the crystal structure of palmitic acid (C16:0) supports this assumption (see below and Fig. 2B). The fatty acids are retained in the binding pocket through (i) the electrostatic interaction between the electronegative carboxylate head group and the elaborate hydrogen bonding network formed by the Gln54 ring and (ii) the hydrophobic interaction existing between the aliphatic tail of the fatty acids and the hydrophobic cavities that exists between Leu37 and Leu51 residues of COMPcc (Figs. 1B and 2A). These hydrophobic cavities can accommodate fatty acids of different lengths within the channel by mediating interactions with the aliphatic side chains. All amino acid residues in positions *a* and *d* of the heptad repeat pattern contribute to van der Waals contacts with the alkyl chain of the bound fatty acids. The terminal methyl groups are held in a fixed position by Thr40 (for C14:0), Leu37-Thr40 (for C16:0) and Leu37 (for C18:0). This interaction is elicited by the longitudinal extension of the fully saturated elongated fatty acids. The C20:0 fatty acid complex is well ordered up to Leu37 after which point the aliphatic tail becomes disordered (data not shown). Based upon this observation we propose that the region Leu37 to Leu51 form the core hydrophobic fatty acid binding region.

**Table 1:** Data collection and refinement data.

Data collection				
Fatty acid Pdb-code	*C14 3V2N*	*C16 3V2Q*	*C18 3V2P*	*C18:1 3V2R*
Resolution range (Å)	20.6-1.8 (1.9-1.8)	24-2.2 (2.32-2.2)	29.4-1.87 (1.99-1.87)	29.4-2.75 (2.92-2.75)
Unique reflections	17542	9799	15795	4761
Completeness %	94.9 (94.3)	99.1 (98.7)	99.3 (95.7)	92.6 (93.0)
Redundancy	2.0	4.4	4.0	2.9
R_sym_ 1	10.1 (31.8)	8.1 (29.6)	7.4 (28.9)	8.3 (32.4)
Cell dimensions (Å,°)				
A	38.35	38.7	37.9	37.86
B	49.39	48.5	48.9	49.11
C	54.82	53.3	53.9	54.25
β	103.86	103.8	104.1	104.14
**Refinement**				
Rfactor^2^ (%), no sigma cutoff	21.2 (26)	20.8 (23.0)	20.7 (28.5)	20.1 (31.3)
R _free_ (%), no sigma cutoff	27.6 (33)	27.5 (31.1)	25.5 (35.7)	27.5 (36.3)
Average B factors (Å^2^)				
Protein/Water/Ligands	26.3/44.3/35.2	48.9/66.1/45.8	29.6/39.3/38.5	41.6/38.5/37.1
Bond length (Å)^3^	0.005	0.006	0.005	0.008
Bond angles (°)^3^	0.9	1.0	0.9	1.1
Ramachandran plot^4^	96.7/2.4/0.5/0.5	95.7/3.3/0/1	97.6/1.5/0/1.0	93.8/5.2/0.5/0.5

Values for the highest resolution shells are in parentheses.

1 R_sym_ = 100 * Σ*I*−<*I*>|/Σ*I*.

2 R_factor_ = Σ||*F*_obs_|−|*F*_calc_|/Σ|*F*_obs_|.

3 Root-mean-square error.

4 Percentage of residues in most favoured/additional allowed/generously allowed and disallowed regions. All datasets were measured at a wavelength of 1.25 Å.

A significant structural difference was observed in the binary COMPcc-oleic acid complex. Oleic acid (C18:1), whose aliphatic tail has a single *cis*-double bond, is held fixed to Leu44 at the *cis*-double bond kink, while the rest of the aliphatic tail remains highly disordered. The opening of the unligated COMPcc channel has been determined to be of maximal width (∼6 Å) at position Val47. This region, therefore, serves to accommodate the *cis*-configured double bond of oleic acid (Fig. 1A) [6]. The sp^2^-hybridized double bond between C9-C10 of oleic acid is thus tightly fitted into a hydrophobic ring of the β-branched side chains of Val47, which is a *d* residue in the heptad repeat pattern of COMPcc. Oleic acids C11 methylene, that immediately follows the double bond, forms van der Waals contacts with three of the five Leu44 side chains in its vicinity.

### Palmitic acid – inside and outside of the pentameric channel system

The structural studies on palmitic acid (C16:0) in complex with COMPcc reveal the presence of two ligand molecules at separate sites (Figure 2). The fatty acid inside the COMPcc channel adopts a linear, elongated conformation with a total length of ∼19 Å (Figure 2A). With its terminal methylene groups, C15–C16, the palmitic acid reaches into the cavity between Leu37 (position *a* in the heptad repeat pattern) and Thr40 (position *d*). Polar interactions of the carboxylate head group with the proposed dipole of the Gln54 ring system stabilize the ligand inside the channel. The overall shape of the electron density map suggests that the ligand molecule is rotating freely inside the channel. This is supported by the fact that a preferred lateral orientation in a stepwise refinement protocol could not be detected (see Materials and Methods). The second palmitic acid molecule is located outside the COMPcc channel and revealed a curved conformation with a kink at position C4–C6 (Fig. 2B). Interestingly, the bent methylene tail is surrounded by a water cloud, and the carboxylate head group is oriented towards a five-membered thioether ring system formed by the Met33 side chains.

### Fluorescence spectroscopy

**Figure 3:**
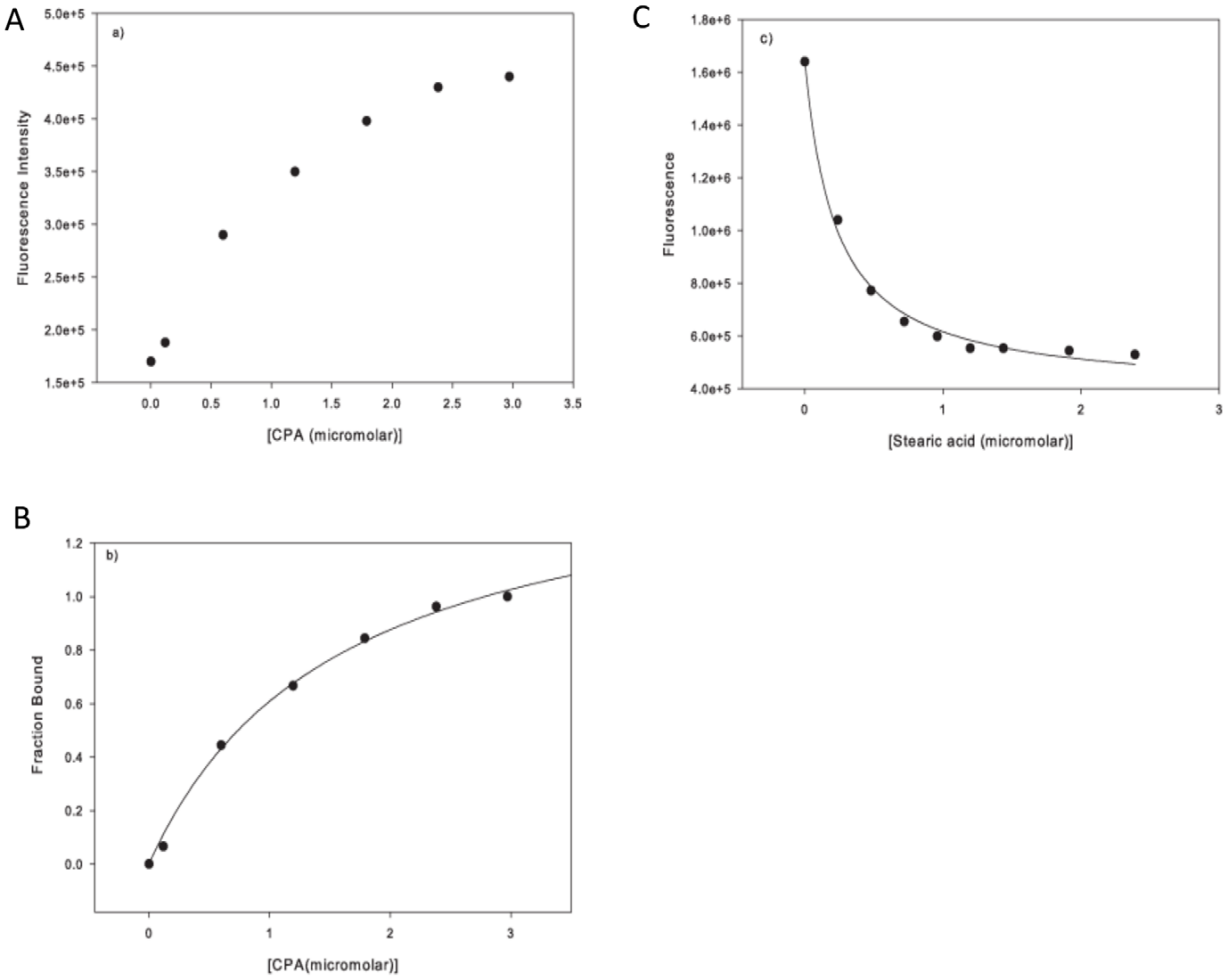
Fluorescence spectra. (**A**) Fluorescence spectrum of CPA as a function of COMPcc concentration in solution, measured at 420 nm. The excitation wavelength was 320 nm and the emission was measured at 420 nm, the slit width were set at 4 nm wavelength band width. (**B**) The fraction of CPA bound to COMPcc follows a hyperbolic one-site binding with a binding constant of K_D_ = 0.7±0.1 µM. (**C**) The effect of fatty acids on the fluorescence of the COMPcc-CPA complex. In this case stearic acid is shown. The decrease in fluorescence follows a hyperbolic trend, with the correlation line used to find [*FA*]_1/2_, the concentration of fatty acid at which the fluorescence singal is half the original.

*Cis-*parinaric acid (CPA) was used as fluorescence probe to investigate the fatty acid binding properties of COMPcc (see also Material and Methods). The free probe has low fluorescence in aqueous solution, however, its fluorescence is increased significantly in the event of protein binding (Fig. 3A). The binding of CPA to the protein follows a simple hyperbolic curve, indicating that one ligand of CPA binds to one molecule of protein. The CPA probe can also be used to characterize the binding of fatty acids to COMPcc (Fig. 3B/C). Titration of fatty acids to the CPA-COMPcc complex will displace CPA leading to a decreased fluorescence. This reduction in the fluorescence signal follows a hyperbolic profile as shown by the correlation line depicted in Fig. 3C. The binding data are summarized in Table 2. The fatty acids all bind strongly to COMPcc, with binding constants in the sub-micromolar range. For elongated, saturated fatty acids, progressively increasing the chain length from C14 to C18 resulted in a decrease in the k_d_ value, indicating stronger binding of the fatty acid to the channel. However, increasing the chain length further to C20 causes an abrupt increase in the k_d_ value, indicating a reduction in binding affinity. The addition of a single double bond to stearic acid almost doubles the k_d_ value, showing that the geometric kink at position C9 in oleic acid (C18:1) disrupts favourable interactions between COMPcc and oleic acid.

**Table 2:** Fluorescence spectroscopy data.

Cargo	[*FA*]_1/2_ (µM)	K_d_ (µM)	Interaction Free Energy (kcal/mol)
C14:0 [Myristic acid]	0.32±0.05	0.8±0.1	−8.31±0.07
C16:0 [Palmitic acid]	0.22±0.02	0.51±0.07	−8.57±0.08
C18:0 [Stearic acid]	0.11±0.03	0.44±0.06	−8.67±0.08
C18:1 [Oleic acid]	0.39±0.05	0.9±0.1	−8.24±0.06
C20:0 [Arachidic acid]	0.28±0.04	0.7±0.1	−8.42±0.08
cis-Parinaric acid	N/A	0.7±0.1	−8.42±0.08
COMPcc vitamin A^1^	N/A	0.6	−8.4
Q54I vitamin A^1^	N/A	0.8	−8.3

All data were repeated in triplicate.

1 [9], [27].

## Discussion

The N-terminal cavity of COMPcc is able to bind different single fatty acid molecules, with their charged carboxylate head group oriented towards the Gln54 ring system and the methylene tail oriented towards the N-terminus. The ability of COMPcc to bind various fatty acid molecules is directly related to its physicochemical properties. A key role in the electrostatic fixation of polarized ligands inside the aliphatic channel is played by the Gln54 ring system. The Gln54 residue belongs to a four amino acid motif (QVKE) that is conserved among the pentameric thrombospondins (TSP-3, TSP-4, and COMP) [26]. Gln54 is situated at position *d* of the characteristic heptad repeat (*a−g*)*_n_*, which is unusual, since the *a* and *d* positions are normally occupied by hydrophobic residues. The hydrogen bonds of the Gln54 ring are arranged into a funnel-like manner, such that the partial positive charges on the amide nitrogens are oriented towards the bottom of the funnel and the partial negative charges on the carbonyl oxygens towards the top. This creates a dipole, which is parallel to the dipole moment of the α-helices. The positively-charged bottom of the funnel can act as a trap for negatively-charged ions, as demonstrated in the native structure of COMPcc where a chloride ion is bound [6]. Interestingly, it was shown that the melting point of COMPcc was increased from 73°C to 104°C when Gln54 was mutated to a Leu residue [27]. This implies an evolutionary advantage of the less thermostable wild type COMPcc over the Q54L mutant and suggests an additional function of the glutamine residues inside the pentameric channel. This decrease in thermal stability can be compensated by ligand binding: the midpoint transition temperature (T_m_) of unfolding increased by 2°C with benzene or cyclohexane bound in the channel, by 8°C when vitamin D_3_ and by 10°C with 18:1 *trans-*9 elaidic acid [9].

**Figure 4:**
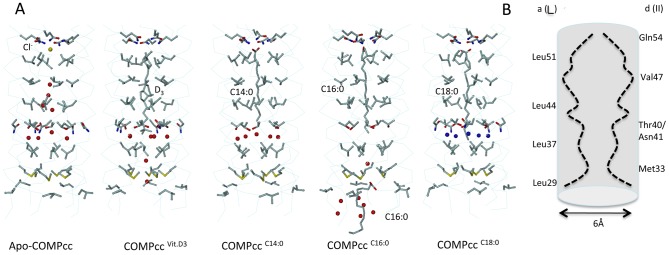
Binding mechanism. (**A**) Structural comparison of apo-COMPcc with the vitamin D_3_ and different fatty acid complexes. The helical backbone is shown as a Cα-trace mode and the individual side chains of amino acid residues in *a* and *d* positions are highlighted. Individual water molecules are drawn as coloured spheres and ligand molecules are labelled accordingly. A comparison of the various energy-minimized COMPcc structures shows that the most energetically favoured state is achieved when a cargo is loaded into the system. For example, the uncomplexed glutamine ring has a ΔG of −6.02 kcal/mol in comparison to −9.8 kcal/mol when a fatty acid is bound. (**B**) Schematic presentation of the variation of the inner diameter along the pentameric COMPcc channel.

Two additional core residues, all at the *d* position of the heptad repeat play a crucial role in the binding of diverse cargo elements (Fig. 4). Firstly, Met33 at the N-terminal opening of the COMPcc channel forms a gating pore with a diameter of 3.4 Å. The CH_3_-moieties face each other and establish strong van der Waals contact forces (Fig. 2B). In contrast the polarizable sulphur components of the thioether are oriented towards the inner core of the pentameric channel. Therefore, one can assume that in order for any ligand to enter the COMPcc channel, the gate has to open thereby permitting access. This assumption is underlined by changes within the helical backbone at the very N-terminus (data not shown). Secondly, Thr40, a subsequent residue in the next heptad repeat, forms interhelical hydrogen bonds between its β-hydroxyl group and the amide group of Asn41. Previous work has shown, that the side chains of the individual Thr40 residues undergo significant re-orientations during ligand binding [8]. In addition to re-orientation, it has also been shown that between the concentric Thr40/Asn41 arrangement and Leu37, a water chamber is formed that contains up to five water molecules inside the pentameric channel (Fig. 4). Comparing wild type COMPcc (pdb-code 1VDF) with COMPcc in complex with vitamin D_3_ (pdb-code:1MZ9), myristic acid (pdb-code:3V2N), palmitic acid (pdb-code:3V2Q) and stearic acid (pdb-code:3V2P) reveals an interesting pattern (Fig. 4). Whereas apo-COMPcc has water molecules lined up along the full length of the channel, the complex structures only contain water in the water chamber (Fig. 4A). An interesting result is observed in the structure of the COMPcc-palmitic acid complex. In this case, the water chamber is empty and instead a cloud of water molecules is surrounding a second bent palmitic acid ligand that is located outside the entrance to the channel (Fig. 2B). This suggests that the release of channel waters plays a key role in facilitating the binding of fatty acids into the pentameric COMPcc channel. To summarize, our observations suggest that the core residues Met33 (gating pore), Thr40/Asn41 (water chamber) and Gln54 (electrostatic trap) are essential components for the binding of fatty acids by COMPcc.

The local environment of the aliphatic tail of the individual fatty acids is characterized by van der Waals contacts with β-branched side chains at *a* and *d* positions, pointing inside the channel of COMPcc (Figs. 1 and 2). The binding site is fully extended, providing space for fatty acids up to ∼22 Å in length (equivalent to C20:0). A careful comparisons of the crystallographic B-factors for the aliphatic tail carbons (C3 to C15) showed that they are similar in magnitude to those of the adjacent side chains of COMPcc. Whereas the methylene tail reveals an averaged B-factor of ∼41 Å^2^, amino acid residues Leu37, Thr40, Leu44, Val47 and Leu51 show an averaged individual B-factor for their side chains of ∼38 Å^2^. These finding suggests nearly fully occupancy of the fatty acid ligands inside the pentameric channel. However, the crystallographic studies on C16:0 at 2.2 Å resolution show a flattened electron density map for the ligand, missing the expected fine contouring for the individual CH_2_-groups (Fig. 2A). This suggests that the ligand is rotating inside the channel. The role of hydrophobic interactions in the binding of nonpolar ligands to COMPcc can be assessed by analyzing how elongation of the fatty acid aliphatic chain affects the binding constant. For example, the binding data indicate that adding two carbons to myristic acid results in a decrease of ∼0.26 kcal/mol in the binding energy (Table 2). This is only a fraction of ∼0.8 kcal/mol, the free energy cost of hydrophobic solvation of the methyl group. A possible explanation for this smaller effect is that the binding of fatty acids to COMPcc is accompanied by a loss of conformational entropy in the aliphatic chain. In other words, when fatty acids bind to COMPcc, the aliphatic chain can not access all its conformational isomers, this entropic loss can partially cancel the gain in free energy due to the hydrophobic effect.

Because myristic acid has 14 carbon atoms, the contribution of the hydrophobic effect (this is equivalent to removing seven or eight pairs of carbon atoms) to the binding of myristic acid to COMPcc can be roughly estimated to be between 2.1 kcal/mol and 2.4 kcal/mol. From the K_d_ value, the free energy of binding of myristic acid to COMPcc can be estimated to be approximately 8.4 kcal/mol. This indicates that the hydrophobic effect contributes about a fourth of the interaction energy of the fatty acid binding.

It must also be emphasized that although COMPcc binding causes a loss of conformational entropy in the fatty acid ligands, the COMPcc binding pocket is still relatively flexible. This flexibility is shown by the fact that COMPcc can accommodate the unsaturated stearic, oleic and CPA molecules with only a modest change in the binding constant.

Coiled-coil proteins such as COMPcc are attractive candidates for the design of drug delivery systems [10], [28], [29], [30]. In this work we have studied the hydrophobic binding pocket of COMPcc and have characterized the various interactions that play an important role in the binding of hydrophobic ligands to the protein. The following is a summary of our findings:

1. The COMPcc channel has been shown to be very flexible and this work demonstrates that the protein can accommodate a wide range of ligand geometric variations in its binding pocket. We suggest that a possible reason for this flexibility is the hydration of COMPcc channel in the apo-state. The presence of internal water molecules allows the coiled-coil to participate in “breathing motions” demonstrated by the dynamic opening of the COMPcc channel to accommodate spacious molecules. This remarkable capability is most dramtically illustrated in the COMPcc - vitamin D_3_ complex, in which the volume of the cavities increases by approximately 30 percent upon binding of the ligand [8]. We intend to study the role of these internal water molecules on the dynamics of the COMPcc channel in future studies.
2. The water chamber as defined by the residues Thr40 and Asn41, seems to play an important role in the ligand binding process. Our results indicate that disrupting the water chamber has an adverse effect on binding. Future work will determine the role of these two residues in establishing the water chamber and elucidate the role of the water chamber in ligand binding.
3. We have quantified the contribution of hydrophobicity to the ligand binding process. In the case of the studied fatty acids, only approximately a fourth of the binding free energy is contributed by the hydrophobic effect and the rest is mostly due to interactions between the carboxylate head group and the Gln54 ring system. COMPcc is an attractive candidate for the design of a Carrier-Pathfinder-System [10]. It combines unique storage properties for otherwise insoluble signalling molecules with the possibility that a targeting molecule can be attached in order to direct it to a specific location for delivery of a target cargo [31], [32].
