# Correction: The Pentameric Channel of COMPcc in Complex with Different Fatty Acids

**DOI:** 10.1371/annotation/65458cd1-ed2b-43f3-a3f8-2bbff27ca87c

**Published:** 2013-05-09

**Authors:** Ainsley McFarlane, George Orriss, Natalie Okun, Markus Meier, Thomas Klonisch, Mazdak Khajehpour, Jörg Stetefeld

The name of the first author was incorrect in the article.

The correct author name is: Ainsley McFarlane

The correct citation is: McFarlane AA, Orriss G, Okun N, Meier M, Klonisch T, et al. (2012) The Pentameric Channel of COMPcc in Complex with Different Fatty Acids. PLoS ONE 7(11): e48130. doi:10.1371/journal.pone.0048130 

